# A Rare Case of Triple Infection with Leptospira, Hepatitis A Virus, and Hepatitis E Virus

**DOI:** 10.3390/reports8040225

**Published:** 2025-10-31

**Authors:** Vasileios Petrakis, Nikoleta Babaka, Maria Panopoulou, Dimitrios Papazoglou, Periklis Panagopoulos

**Affiliations:** 1 Department of Infectious Diseases, 2nd University Department of Internal Medicine, University General Hospital Alexandroupolis, Democritus University of Thrace, 68100 Alexandroupolis, Greece; mpampakanikoleta@gmail.com (N.B.); dpapazog@med.duth.gr (D.P.); ppanago@med.duth.gr (P.P.); 2 Laboratory of Microbiology, University General Hospital Alexandroupolis, Democritus University of Thrace, 68100 Alexandroupolis, Greece; mpanopou@med.duth.gr

**Keywords:** Hepatitis A Virus, Hepatitis E Virus, Leptospirosis, coinfections, zoonotic diseases

## Abstract

**Background and Clinical Significance**: Simultaneous, multiple infections coinfections caused by zoonotic or fecal-orally transmitted diseases are common in tropical and subtropical regions. Published data report that leptospirosis may coexist with other infections, complicating the clinical presentation and trajectory due to overlapping symptoms and leading to more severe clinical progress. **Case Presentation**: We describe a clinical case of a 34-year-old female diagnosed with a triple infection caused by Leptospira, Hepatitis A Virus, and Hepatitis E Virus. To our knowledge, this is the first case described in the literature in a non-endemic area without travel history to tropical or subtropical regions. The patient presented with one-week history of influenced clinical status, myalgia, headaches, nausea, high fever, bloody diarrheas, and abdominal pain. During the last two days, she also developed jaundice. Swimming in the rock pools of the island where she lives was indicated as the source of the infection. The laboratory tests revealed increased values of inflammatory markers, thrombocytopenia, and severe abnormalities of liver function. Serological and PCR tests for a wide range of pathogens proved an acute infection caused by *Leptospira interogans*, Hepatitis A virus, and Hepatitis E Virus. The patient received intravenous fluids and antibiotic treatment with ceftriaxone for seven days leading to gradual clinical improvement and normalization of liver function tests with subsequent reduction in jaundice within 30 days. **Conclusions**: This case report suggests that clinical suspicion and laboratory investigation should include the probability of coinfections even in non-endemic areas based on medical history of the patients. An early diagnosis of a zoonotic disease and other infective agents of acute hepatitis are vital in order to choose the appropriate treatment option and avoid severe complications.

## 1. Introduction and Clinical Significance

Leptospirosis is a globally distributed zoonotic disease caused by pathogenic spirochetes of the genus Leptospira [1]. It is a greatly underreported disease, particularly in tropical regions [1]. Attempts at surveillance programs suggest that it may be the most common zoonosis [1]. The major pathogenic Leptospira for humans is *Leptospira interogans*, an aerobic, Gram-negative bacterium [2]. In scanning electron micrographs, *Leptospira* species show helical structure, curved ends, and motility due to two axial flagella underlying the membrane sheath, which are inserted at opposite ends of the cell and extend toward the central region [2]. Leptospirosis is endemic throughout the world. Although outbreaks may follow every period of excess rainfall, the peak incidence occurs in the rainy season in tropical regions and the late summer to early fall in temperate regions [3]. Leptospirosis is maintained in nature by chronic renal infection of carrier animals [3]. The most important reservoirs are rodents and other small mammals, but livestock and companion animals are also significant sources of human infection [1,2,3]. The clinical spectrum varies from an asymptomatic disease or a self-limited, without-fever illness to severe manifestations such as Weil’s disease with jaundice, renal failure and hemorrhages, and severe pulmonary hemorrhagic syndrome [1,2,3]. Socioeconomic factors contribute to the risk of acquiring the disease [4,5,6,7]. Close contact with garbage and sewage at urban slums, low socioeconomic status, low educational level, and conditions such as inadequate access to safe drinking water and sanitation services increase vulnerability to infection [4,5,6,7,8,9]. Leptospirosis is not only an occupational disease associated with activities such as mining, sewer maintenance, livestock farming, and butchering [8,9,10]. In developed countries, many cases occur due to slum living, recreational activities involving immersion in water, and occupational exposure in agricultural activities [10,11,12]. Proximity to pig farms, low income, outdoor occupations, and inadequate health education increase the risk of leptospirosis [10,11,12].

In tropical and subtropical regions, leptospirosis frequently coexists with other infections such as dengue fever, malaria, and Epstein–Barr virus which could complicate the clinical presentation and trajectory due to overlapping symptoms and laboratory findings and lead to more severe clinical progress and outcomes [13]. There are also published cases with coinfections of leptospirosis with Hepatitis A virus (HAV) or Hepatitis E virus (HEV), two positive-sense, single-stranded RNA viruses [14,15,16]. These cases occurred in settings with poor sanitation, contaminated water, and concurrent exposure to animal reservoirs. In adults, HEV and HAV infections are leading causes of acute viral hepatitis with worldwide distribution [17,18]. As fecal-orally transmitted viruses, the virus first enters the host via the gastrointestinal tract and replicates in intestinal epithelial cells, and, subsequently, enters the bloodstream via viremia and reaches its target organ, the liver [17,18]. Waterborne outbreaks due to HAV, usually associated with sewage-contaminated or inadequately treated water, are infrequent [17,18]. Dual infection of HAV and HEV is common, because their entry points are similar and could lead to serious complications and increased mortality due to the high risk of acute liver failure in both children and adults [17,18]. However, clinical cases with triple infection of leptospirosis, HAV, and HEV have not been described in the literature.

We report a rare case of a patient in a non-endemic area, without travel history in tropical regions, hospitalized in our department due to jaundice, multiple diarrheas, fever, and severe abnormalities in liver function tests. The diagnostic process concluded to the diagnosis of a triple infection with leptospirosis, HAV, and HEV. The probability of a coinfection should be investigated based on the high hepatotoxic effect revealed in clinical and laboratory findings, including in the differential diagnosis pathogens with common transmission mode and target organs.

## 2. Case Presentation

A 34-year-old female was presented at the Emergency Department due to one-week history of influenced clinical status, myalgia, headaches, drowsiness, nausea, loss of appetite, high fever (>39 °C), bloody diarrheas (more than six episodes per day), and diffuse abdominal pain. During the last two days she also developed jaundice. The patient had no past medical history and did not receive chronic medication. She was not a former or current smoker and did not consume alcohol. She had no travel history in tropical or subtropical regions, but she lives without other family members in a rural area of Samothrace, a Greek island in Northern Aegean Sea within the Evros regional unit of Thrace with low rates of confirmed cases of foodborne or waterborne infections. She has completed her master studies in computer sciences and has achieved a high income and completely remote works from home. She had pets and livestock animals and, according to the medical history, frequently swims in the rock pools of the island. On admission, her vitals assessment revealed fever (39.5 °C), tachycardia (118 beats per minute), low blood pressure (86/62 mm Hg), and normal oxygen saturation (SpO_2_ 98%, FiO_2_ 21%). Cardiopulmonary and lymph node assessment was normal. The physical examination of the abdomen revealed increased abdominal sounds, hepatomegaly without palpable spleen, and diffuse abdominal pain with rebound tenderness. She had jaundice of the conjunctiva and skin. The neurological examination was normal without fecal deficits. Initial laboratory tests revealed an increased count of white blood cells (leukocytes 17.250/μL), normal count of red blood cells (hematocrit 36%), and thrombocytopenia (106.000/μL). The liver function tests were extremely abnormal with excessive increase in cholestatic enzymes and aminotransferases (alkaline phosphatase 333 U/L, gamma-glutamyl transferase 98 U/I, aspartate aminotransferase 684 U/L, alanine aminotransferase 991 U/L, total bilirubin 10.9 mg/dL, and direct bilirubin 10.4 mg/dL). The value of creatinine was normal (0.8 mg/dL). Inflammatory markers were significantly increased. The values of C-reactive protein were 94 mg/dL, of Erythrocyte Sedimentation Rate (ESR) 63 mm/h and of procalcitonin 0.6 ng/mL. The pregnancy test was negative and she did not mention history of menstrual cycle deterioration.

**Figure 1:**
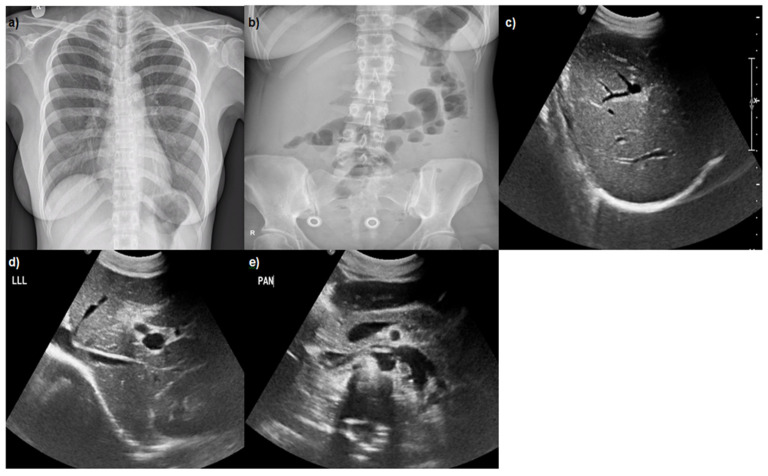
(**a**) Normal chest X-ray; (**b**) abdominal X-ray: mild air–fluid levels; (**c**–**e**) abdominal ultrasound: hepatomegaly, wall-thickening of gall bladder without stones, ‘starry sky’ appearance of parenchyma, and findings of hepatitis.

Abdominal ultrasound was performed, indicating hepatomegaly, wall-thickening of gallbladder without stones, ‘starry sky’ appearance of parenchyma, and findings of hepatitis (Figure 1). Blood and urine cultures were negative. Due to the bloody diarrheas, stool cultures and parasitic examinations were evaluated in three specimens which were negative. There was high clinical suspicion based on medical history for fecal-orally transmitted pathogens, zoonotic diseases, and causes of hepatitis syndrome. Examination for infective agents associated with bloody stools including Enterohemorragic Escherichia coli, Cambylobacter, Salmonella, Shigella, Yersinia, Entamoeba, and Schistosoma species was negative. Wright test for Brucella and Turbeculin skin testing were negative. Screening for Human Immunodeficiency Virus, Hepatitis B Virus, Hepatitis C Virus, Malaria, West Nile virus, Cytomegalovirus, and Epstein–Barr virus were all negative. The serological tests revealed acute infection caused by *Leptospira interogans*, HAV, and HEV (positive titers of IgM antibodies and negative IgG antibodies). These diagnoses were confirmed with positive PCR blood tests for HAV and HEV and with positive PCR blood and urine tests for leptospirosis in the Laboratory of Microbiology of our hospital, but also in National Microbiology Reference Laboratory. Waterborne transmission of the infections was supported by the habit of the patient swimming in the rock pools of the island.

**Figure 2:**
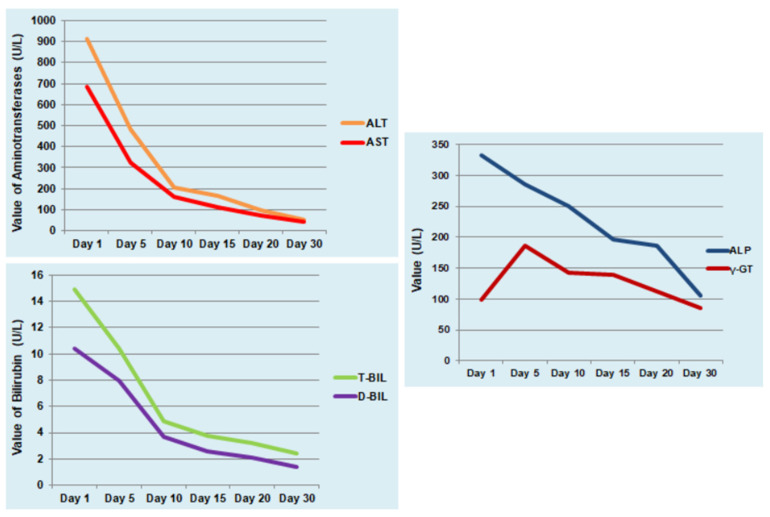
Progression of liver function tests during hospitalization and at follow-up visits. Alanine aminotransferase (ALT, U/L), aspartate aminotransferase (AST, U/L), alkaline phosphatase (ALP, U/L), gamma-glutamyl transferase (γ-GT, U/L), total bilirubin (T-BIL, mg/dL), and direct bilirubin (D-BIL, mg/dL).

Based on the findings of abdominal clinical examination, the patient initially received empirical antibiotic treatment with ceftriaxone and metronidazole and intravenous fluids. After the diagnosis of leptospirosis, the ceftriaxone was maintained for seven days. The patient remained without fever after the fourth day of hospitalization and the abdominal pain and episodes of bloody diarrheas were completely resolved after six days. Liver function tests were gradually normalized with subsequent reduction in jaundice within 30 days. Although the patient had a triple infection by three hepatotoxic pathogens, she did not develop severe complications of leptospirosis such as hemorrhages and severe Weil’s disease or acute liver failure. The laboratory findings and clinical course of the patient are shown in Table 1 and Figure 2. Positive titers of IgG antibodies were detected for leptospirosis at the tenth day after hospital admission and for HAV and HEV at the fourteenth day. The infection by three different pathogens raised the suspicion of immunodeficiency which was excluded after normal serological, virological, and immunological testing and absence of abnormal findings in Computed Tomography of thorax and abdomen.

## 3. Discussion

Simultaneous, multiple infections are not uncommon in immunocompromised people and have been increasingly described after the HIV/AIDS pandemic [19]. Coinfections caused by zoonotic or fecal-orally transmitted diseases are also common in tropical and subtropical regions [14,15,16,17]. However, we report a rare clinical case of an immunocompetent female without travel history diagnosed with a triple infection caused by leptospirosis, HAV, and HEV in a non-endemic area which has not been documented in the literature.

There are a few published reports about clinical cases of leptospirosis with other coinfections. Varga et al. published a case report of a young male patient with triple infection with HAV, Epstein–Barr virus, and Leptospira with symptoms onset two weeks after holidays in the countryside of Romania [14]. The 23-year-old patient with no other known medical condition presented with a 5-day history of influenced clinical status, myalgia, headaches, drowsiness, high fever, cervical adenopathy, and jaundice [14]. The laboratory examinations revealed severe hepatic cytolysis syndrome [14]. After the positive lab tests, the patient received intravenous (IV) antibiotic treatment initially with amoxicillin/clavulanic acid which was modified due to persistent symptoms to ceftriaxone leading to significant clinical improvement [14]. Damodar et al. reported another rare case of a 48-year-old female from a rural area in India infected by Hepatitis B and leptospirosis with high titers of *Salmonella paratyphi A* and scrub typhus [15]. She had a 2-week history of high-grade fever with chills, epigastric pain, and cough with findings of jaundice, conjunctival suffusion, pedal edema, and facial puffiness revealed in physical examination [15]. Due to clinical deterioration with severe renal failure, the patient was intubated and died one month after admission, although she received antibiotic treatment with ceftriaxone and subsequently with crystalline penicillin and doxycycline [15]. A clinical case published by Ferraz et al. described a 47-year-old man with known HIV infection who injected drugs diagnosed with acute HCV infection and leptospirosis [16]. The patient developed a self-limited acute febrile hepatitis syndrome with rapid clinical improvement [16]. The HAV/HEV dual infection is common in developing countries, may be associated with outbreaks, and has a questionable impact on clinical progress and outcome of acute hepatitis [20]. In our report case, the HAV/HEV dual infection did not affect the prognosis despite the triple coinfection with leptospirosis and no severe complication was developed.

During the last 20 years in Greece, sporadic cases of leptospirosis have been reported, peaking during the summer months with an average annual incidence of 0.22 cases per 100,000 population [21,22]. The highest average annual frequency incidence of the disease was reported in the region of the Ionian Islands [21]. The majority of patients were involved in agricultural activities [22]. Hepatitis A in Greece has shown a declining overall incidence since 2007, but remains a public health concern in vulnerable populations such as refugees and migrants [23]. Data on Hepatitis E epidemiology in Greece is less comprehensive, with surveillance being challenging due to limited availability of testing and more data available on high-risk groups like hemodialysis patients and liver transplant recipients [24].

## 4. Conclusions

This case report highlights the need to include in our diagnostic process the rare probability of multiple, simultaneous infections by pathogens with similar transmission routes. Coinfections caused by zoonotic diseases and fecal-orally transmitted and hepatotoxic viruses may be more common in tropical regions, but they could also be diagnosed in non-endemic areas without travel history or usual socioeconomic risk factors. An early diagnosis is vital in order to administrate the appropriate treatment such as antibiotics in the case of leptospirosis and achieve the best clinical outcome.

The major learning points are

- Leptospirosis is a globally distributed zoonotic disease caused by pathogenic spirochetes of the genus *Leptospira*. It is a greatly underreported disease, particularly in tropical regions.
- In tropical and subtropical regions, leptospirosis frequently coexists with other infections and zoonotic diseases which could complicate the clinical progression and outcome due to overlapping symptoms and laboratory findings.
- A broad serological testing based on the medical history in cases of acute hepatitis syndrome should be conducted, while multiple, simultaneous infections could exist even in non-endemic areas. An early diagnosis and treatment of each pathogen is crucial in order to improve clinical progress and outcome.

## Figures and Tables

**Table 1:** Laboratory findings during hospitalization and at follow-up visits.

	Normal Ranges *	Day 1	Day 2	Day 3	Day 4	Day 5	Day 6	Day 7	Day 15	Day 30
**Leucocytes**	4000–10,000/µL	**17,200**	**17,940**	**13,450**	**10,900**	7350	6400	6580	5800	5320
**Lymphocytes**	25–40%	**11.7**	**8.8**	**9.0**	**19.2**	**19.9**	26.1	27.8	35.7	38.9
**Monocytes**	5.5–10%	**4.7**	**4.4**	6.5	7.1	7.9	**11.0**	**14.3**	7.3	4.9
**Neutrophils**	55–65%	**83.6**	**86.2**	**81.8**	**70.8**	**67.0**	56.2	**54.8**	**52.8**	56.2
**Platelets**	150–450 × 10^3^/L	**106**	**98**	**109**	**126**	**137**	159	162	235	258
**Prothrombin time**	9.4–12.1 s	11.8	**12.4**	**13.0**	11.2	10.3	10.1	10.5	9.8	10.2
**ALT**	0–40 U/L	**911**	**931**	**728**	**661**	**481**	**398**	**203**	**108**	**53**
**AST**	0–41 U/L	**684**	**703**	**505**	**463**	**324**	**205**	**144**	**98**	**46**
**ALP**	30–120 U/L	**333**	**452**	**378**	**399**	**285**	**267**	**255**	**192**	**108**
**γ-GT**	7–32 U/L	**98**	**167**	**205**	**197**	**186**	**191**	**159**	**98**	**79**
**Total Bilirubin**	0–1.2 mg/dL	**14.9**	**16.1**	**13.56**	**12.13**	**10.38**	**8.29**	**5.76**	**3.2**	**1.82**
**Direct Bilirubin**	0–0.2 mg/dL	**10.4**	**11.38**	**9.98**	**8.66**	**7.94**	**5.85**	**3.26**	**1.15**	**0.96**
**Creatinine**	0.6–1.2 mg/dL	0.8	0.89	0.79	0.66	0.62	0.6	0.59	0.61	0.57
**CRP**	<10 mg/L	**94**	**92**	**58**	**33**	7	3	1	0.1	0.1
**Procalcitonin**	<0.5 ng/mL	**0.6**	**0.9**	**1**	**0.5**	0.4	0.3	0.2	0.0	0.0

* Normal values provided by Laboratory of Microbiology of University General Hospital of Alexandroupolis. Abnormal values are highlighted in bold.

## Data Availability

The research data are available after applying to the corresponding author. The data are not publicly available due to privacy concerns.
